# *TP53* Arg72 as a favorable prognostic factor for Chinese diffuse large B-cell lymphoma patients treated with CHOP

**DOI:** 10.1186/s12885-017-3760-0

**Published:** 2017-11-10

**Authors:** Yalu Liu, Xiaogan Wang, Ning Ding, Lan Mi, Lingyan Ping, Xuan Jin, Jiao Li, Yan Xie, Zhitao Ying, Weiping Liu, Chen Zhang, Lijuan Deng, Yuqin Song, Jun Zhu

**Affiliations:** 1 Key Laboratory of Carcinogenesis and Translational Research (Ministry of Education), Department of Lymphoma, Peking University Cancer Hospital & Institute, 52 Fucheng Road, Haidian District, Beijing, 100142 People’s Republic of China; 2 Key Laboratory of Carcinogenesis and Translational Research (Ministry of Education), Peking University Cancer Hospital & Institute, 52 Fucheng Road, Haidian District, Beijing, 100142 People’s Republic of China; 3 Department of Internal Medicine Oncology, Peking University First Hospital, 8 Xishiku Road, Xicheng District, Beijing, 100034 People’s Republic of China

**Keywords:** Diffuse large B-cell lymphoma, *TP53* Arg72Pro, Chop, Rituximab, Prognosis

## Abstract

**Background:**

*TP53* Arg72Pro (SNP rs1042522) is associated with risk of non-Hodgkin lymphoma (NHL). Diffuse large B-cell lymphoma (DLBCL) is the most common subtype of NHL. However, the relationship between this SNP and prognosis of DLBCL in Asians is unknown.

**Methods:**

Genotyping of *TP53* Arg72Pro was done in 425 Chinese DLBCL patients. Two hundred and eighty-nine patients were treated with R-CHOP, and 136 patients received CHOP or CHOP-like as frontline regimen. Three hundred and ninety-six patients were assessable for the efficacy.

**Results:**

Patients with Arg/Arg and Arg/Pro at codon 72 of *TP53* had a higher complete response rate (61% vs. 44%, *P* = 0.007) than those with Pro/Pro. In the subgroup treated with CHOP or CHOP-like therapy, patients with Arg/Arg and Arg/Pro showed a higher 5-year overall survival (OS) rate than those with Pro/Pro (68.8% vs. 23.2%, *P* = 0.001). Multivariate Cox regression analysis revealed *TP53* Arg72 as a favorable prognostic factor in this group. However, the combination of rituximab with CHOP significantly increased the 5-year OS rate of patients with Pro/Pro to 63%.

**Conclusion:**

This study revealed *TP53* Arg72 as a favorable prognostic factor for Chinese DLBCL patients treated with CHOP or CHOP-like as frontline therapy.

## Background

The common *TP53* single nucleotide polymorphism (SNP) rs1042522 (c. 215G > C), results in the substitution of proline (Pro) for arginine (Arg) at codon 72 in the proline-rich domain. p53 Arg72 is more potent in apoptosis induction whereas p53 Pro72 is better in inducing cell cycle arrest and DNA damage repair [1–4].

Several reports demonstrated that *TP53* Arg72Pro was associated with tumorigenesis and clinical outcomes [5–8]. Several meta-analyses of this SNP in cancer risk revealed that the *TP*53 Arg72Pro polymorphism is associated with an increased risk of cancer. In the subgroup analysis, significantly increased cancer risk was observed among Asians in homozygous and recessive models, while in Americans increased cancer risk was observed only in dominant and recessive models [9, 10]. Moreover, a significantly increased non-Hodgkin lymphomas (NHL) risk was found in carriers of the *TP*53 72Pro allele, including in Chinese patients [11–14]. However, the association of *TP53* Arg72Pro with clinical outcomes and prognosis in lymphoma is still uncertain [15, 16].

DLBCL is the most common subtype of NHL [17]. Combined treatment of rituximab and chemotherapy has resulted in improved clinical outcomes [18–21]. However, one-third of responding patients become refractory to treatment and no-responders to second line therapy or immune-chemotherapy-based third line therapy [22, 23]. To evaluate the influence of *TP53* Arg72Pro on the prognosis of NHL in the Chinese population, this retrospective study was done in 425 DLBCL patients treated with CHOP or CHOP plus rituximab (R-CHOP) therapy.

## Methods

### Patients population and response evaluation

The clinical research protocol was approved by the Institutional Review Board and the Ethical Committee of Peking University School of Oncology, Beijing, China. All patients participating in this study signed the informed consent.

Four hundred and twenty-five patients with DLBCL confirmed by our Department of Pathology according to the World Health Organization classification were included in this study. Of the patients, 289 received rituximab in combination with a chemotherapy regimen between January 2000 and January 2015 at the Beijing Cancer Hospital, Peking University School of Oncology. Another 136 patients received CHOP or CHOP-like therapy (e.g. COP, CCOP, CHO or CHOPE) as the frontline chemotherapy. Based on the expression levels of Bcl-6, CD10, and MUM-1 measured by immunohistochemistry, cases were subdivided into germinal center B-cell (GCB) and non-GCB types according to the Hans algorithm [24, 25]. The response to chemotherapy was evaluated after completion of 2 to 3 courses of therapy and 1 to 2 months after completion of all treatment plans, then every 3 months for the first year and every 6 months thereafter until progression.

Overall survival (OS) was calculated from the date of disease confirmation to the date of last follow-up or death. Progression free survival (PFS) was identified as the period between the disease confirmation and progression (relapse and refractory) or disease-related death. Disease status was evaluated via clinical findings and computed tomography and classified as complete response (CR), unconfirmed complete response (CRu), partial response (PR), stable disease (SD), progressive disease or relapse according to the revised response criteria for malignant lymphoma [26, 27]. Patients who had heterozygous (GC) or homozygous G (GG) genotype of *TP53* SNP rs1042522 were designated as G carriers.

### DNA extraction and genotyping

Genomic DNA was extracted from whole blood using the Whole Blood Genome DNA isolation Kit according to the manufacturer’s instructions (Qiagen, Nussloch, Germany). DNA was diluted in AE buffer to a final stock concentration of 20 ng/μl, and 2 μl was used in each PCR reaction.

Sanger chain termination sequencing was used to determine the *TP53* SNP rs1042522 genotype. PCR product was amplified using forward 5’TTGCCGTCCCAAGCAATGGATGA3’ and reverse 5’TCTGGGAAGGGACAGAAGATGAC3’ primers.

Following an initial denaturation step at 94 °C for 3 min, amplification was carried out by 40 cycles of denaturation at 94 °C for 30s, annealing at 62 °C for 40s, and extension at 72 °C for 40s. This was followed by a final extension at 72 °C for 5 min. Amplified products were analyzed by gel electrophoresis on 1.5% agarose gels and were sequenced using an ABI 3730XL Avant Genetic Analyzer (Applied Biosystems Inc., USA). Determination of the *TP53* rs1042522 genotype was achieved blindly on coded specimens by Sanger chain termination sequencing with the Seqman software (DNASTAR, USA).

### Statistical analysis

All statistical analysis was carried out using SPSS software for Windows (version 19.0). An effect was considered statistically significant at *P* < 0.05. Genotype frequencies and clinical parameters were compared using a χ2 test. The Kaplan–Meier method was used to construct survival curves, and results were compared using a log-rank test. Multivariate Cox regression analyses were used to assess associations between survival time and potential risk factors. The Hardy–Weinberg equilibrium was used to test for deviation of allele and genotype frequency.

## Results

### Patients’ characteristics

The general characteristics of the 425 DLBCL patients (175 male and 250 female) in this study are summarized in Table 1. The median age at diagnosis was 54 years (range, 15–90 years). Two hundred and forty-three (57.2%) patients were in stage 3 or 4, and 136 (32.0%) patients had intermediate-to-high or high international prognostic index (IPI) scores. One hundred and twelve (26.4%) patients were classified into GCB subgroup, 251 (59.1%) patients were classified into non-GCB subgroup, and 62 patients had incomplete records. Two hundred and thirty-four (55.1%) patients exhibited B symptoms at diagnosis and 109 (28.2%) patients showed an elevated β_2_-MG level. Two hundred and eighty-nine (68%) patients were treated with R-CHOP therapy and 136 patients were treated with CHOP or CHOP-like therapy only.

**Table 1:** DLBCL patients’ characteristics and correlations with *TP53* SNP rs1042522

Clinical parameters	No.	Genotype		*P*	Clinical parameters	No.	Genotype		*P*
		GG + GC	CC				GG + GC	CC	
Gender					β_2_-MG				
Male	175	147	28	0.213	Positive	109	79	30	0.004
Female	250	198	52		Negative	277	236	41	
Age					LDH				
≤60	269	216	53	0.543	Positive	192	155	37	0.83
>60	156	129	27		Negative	233	190	43	
Stage					ESR				
I-II	182	146	36	0.662	Positive	230	185	45	0.501
III-IV	243	199	44		Negative	143	119	24	
IPI score					ECOG score				
0–2	289	231	58	0.338	0–2	368	296	72	0.32
3–5	136	114	22		3–4	57	49	8	
Subtype					HBV infection				
GCB	112	92	20	0.709	Positive	200	159	41	0.355
Non-GCB	251	202	49		Negative	218	181	37	
B symptoms					Treatment				
Positive	234	200	34	0.012	CHOP/CHOP-like	136	107	29	
Negative	191	145	46		R-CHOP	289	238	51	0.366

*IPI* International prognostic index, *GCB* Gernminal center B cell subtype, *MG* Microglobulin, *LDH* Lactate dehydrogenase, *ESR* Erythrocyte sedimentation rate, *ECOG* Eastern cooperative oncology group, *HBV* Hepatitis B virus

### *TP*53 SNP rs1042522 in 425 DLBCL patients

We detected the genotype of *TP*53 SNP rs1042522 in 425 patients. As shown in Table 2, 28% patients carried the homozygous GG genotype (Arg/Arg), 53.2% patients had the heterozygous GC genotype (Arg/Pro), and18.8% patients carried the homozygous CC genotype (Pro/Pro). The frequency of the G allele in 425 patients was 55%, and the frequency of the C allele was 45%. The genotype distribution of SNP rs1042522 in the DLBCL population analyzed in this study was in Hardy-Weinberg equilibrium (*P* = 0.135), and the allele distribution was close to the frequency distribution seen in the Asian population based on the dbSNP database.

**Table 2:** Genotype and allele frequency of *TP53* SNP rs1042522 in 425 Chinese DLBCL patients

Genotype	Frequency	Count
GG (Arg / Arg)	0.28	119
GC (Arg / Pro)	0.532	226
CC (Pro / Pro)	0.188	80
Allele	Frequency	Count
G (Arg)	0.55	464
C (Pro)	0.45	386

### Correlations between SNP rs1042522 and clinical features of DLBCL patients

As shown in Table 1, patients with genotype GG and GC of SNP rs1042522 had a lower positive rate for β_2_-MG than those with genotype CC (25.1% vs. 42.3%, *P* = 0.004). Although the G allele carriers showed a higher positive rate for B symptoms (58.1% vs. 42.5%, *P* = 0.012), the univariate analysis revealed that B symptoms is not an independent prognostic factor for overall survival (*P* = 0.983). The genotype distribution in CHOP or CHOP-like and R-CHOP treated subgroups is unbiased.

### Clinical response according to the genotype of *TP53* SNP rs1042522

Of the 396 patients evaluable for response to CHOP or CHOP-like therapy with or without rituximab, the OR rate was 84.1% (333 of 396 patients), including a CR rate of 57.8% (229 of 396 patients) and a PR rate of 26.3% (104 of 396 patients). As shown in Table 3, of the 396 patients, patients with genotypes GG and GC exhibited higher CR and OR rates than those with the genotype CC (61% vs. 44%, *P* = 0.007; 86% vs. 76%, *P* = 0.033). The combination of rituximab in treatment significantly increased the CR rate (65% vs. 38%, *P* < 0.001). In the subgroup treated without rituximab, a relatively higher CR rate was achieved in patients with genotype GG and GC than in those with genotype CC (45.78% vs. 12.5%; *P* = 0.004). However, this significant difference vanished in the subgroup treated with combination of rituximab. In the subgroup treated with R-CHOP or R-CHOP-like, patients with genotypes GG and GC exhibited similar CR (66.4% vs. 58.8%, *P* = 0.304) rates and OR (87% vs. 76.5%, *P* = 0.056) rates to those with the CC genotype.

**Table 3:** Clinical response according to the genotype of *TP53* SNP rs1042522

Response	Genotype		*P*
	GG + GC (%)	CC (%)	
All patients			
CR	196(61)	33(44)	0.007
PR + PD + SD	125(38.9)	42(56)	
OR	276(86)	57(76)	0.033
PD + SD	45(14)	18(24)	
Patients without Rituximab			
CR	38(45.8)	3(12.5)	0.004^a^
PR + PD + SD	45(54.2)	21(87.5)	
OR	69(83.1)	18(75)	0.368
PD + SD	14(16.9)	6(25)	
Patients with Rituximab			
CR	158(66.4)	30(58.8)	0.304
PR + PD + SD	80(33.6)	21(41.2)	
OR	207(87)	39(76.5)	0.056
PD + SD	31(13)	12(23.5)	

*CR* Complete response

*PR* Partial response

*PD* Progression disease

*SD* Stable disease

*OR* Overall response

^a^:Fisher’s Exact Test

### Survival analyses according to the genotype of *TP53* SNP rs1042522

**Fig. 1:**
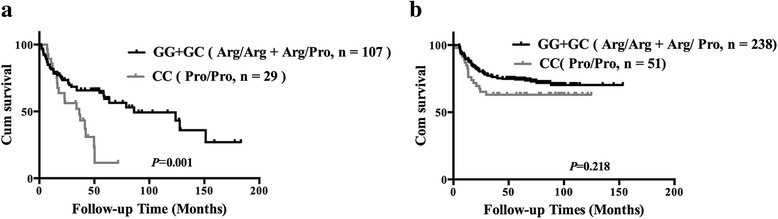
Kaplan-Meier curve of overall survival according to the genotype of *TP*53 Arg72Pro. **a** 136 patients treated with CHOP or CHOP-like therapy. **b** 289 patients treated with R-CHOP therapy

**Fig. 2:**
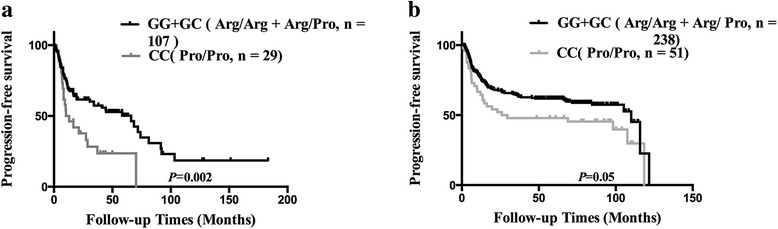
Kaplan-Meier curve of progression free survival according to the genotype of *TP*53 Arg72Pro. **a** 136 patients treated with CHOP or CHOP-like therapy. **b** 289 patients treated with R-CHOP therapy

All 425 patients were evaluated for OS and PFS. After a median follow-up time of 56.23 months (range, 0.83–183.23 months), two hundred and fifty (58.8%) patients relapsed or progressed, 135 (31.8%) patients died and 40 (9.4%) patients lost follow-up. Patients with genotypes GG and GC had a median OS of 57.6 months and a median PFS of 49.7 month respectively, while patients with the genotype CC showed a median OS of 39.9 months and a median PFS of 18.1 months. In the subgroup treated with CHOP or CHOP-like therapy (Fig. 1), patients with genotype GG and GC had higher 5-year OS and PFS rates than those with genotype CC (68.8% vs. 23.2%, *P* = 0.001; 56.1% vs. 25.4%, *P* = 0.002, respectively). However, the integration of rituximab in treatment significantly increased the 5-year OS and PFS rates (57.1% vs. 72.8%, *P* = 0.001; 49.4% vs. 61.3%, *P* = 0.017) in the overall population. Therefore, in the subgroup treated with R-CHOP therapy (Fig. 2), the 5-year OS and PFS rates of CC patients are only about 10% lower than those of G allele carriers (63.0% vs. 74.9%, *P* = 0.218; 51% vs. 63.5%, *P* = 0.05) and did not reach the statistical significance.

### Multivariate analyses

Multivariate analyses were done to evaluate the following variables on OS: age (≤60 vs. >60 years), stage (stages I-II vs. III-IV), IPI score (0–2 vs. 3–5), subtype (GCB vs. Non-GCB), β_2_-MG (positive vs. negative), LDH (positive vs. negative), ESR (positive vs. negative), ECOG score (0–2 vs. 3–4), treatment (CHOP/CHOP-like vs. R-CHOP), and the genotype of *TP53* SNP rs1042522 (GG + GC vs. CC). As shown in Table 4, along with known baseline predictors, treatment with rituximab was confirmed as a favorable prognostic factor (*P* < 0.001, HR = 0.377, 95% CI = 0.222–0.521). Interestingly, focusing only on patients treated without rituximab (Table 5), multivariate analysis revealed the G allele of *TP53* SNP rs1042522 (*TP53* Arg72) as a favorable prognostic factor (*P* = 0.002, HR = 0.343, 95% CI = 0.173–0.679).

**Table 4:** Multivariate analysis of *TP53* SNP rs1042522 on survival in 425 DLBCL patients

Variable	Hazard ratio	95% CIs	*P*
Age	1.645	1.100–2.461	0.015
GCB/Non-GCB	0.444	0.266–0.741	0.002
Stage	2.781	1.681–4.602	0.000
LDH	1.886	1.221–2.913	0.004
β_2_-MG	2.148	1.421–3.246	0.000
R-CHOP/CHOP	0.337	0.222–0.521	0.000

**Table 5:** Multivariate analysis of rs1042522 on survival in 136 patients treated with CHOP or CHOP-like therapy

Variable	Hazard ratio	95% CIs	*P*
GCB/Non-GCB	0.316	0.158–0.633	0.001
IPI score	4.218	2.235–7.962	0.000
GG + GC/CC	0.343	0.173–0.679	0.002

## Discussion

In this study, a retrospective analysis was done to evaluate the influence of *TP53* Arg72Pro on the prognosis of 425 Chinese DLBCL patients treated with CHOP or R-CHOP therapy. Patients with genotype GG (Arg/Arg) and GC (Arg/Pro) of SNP rs1042522 had a lower positive rate for β_2_-MG and higher CR and OR rates for treatment than those with genotype CC (Pro/Pro). In the subgroup treated without rituximab, a significant higher CR rate and higher 5-year OS and PFS rates were achieved in patients with Arg/Arg and Arg/Pro than in those with Pro/Pro. Multivariate analysis revealed *TP*53 Arg72 as a favorable prognostic factor in this group. As the integration of rituximab in treatment significantly increased the CR, 5-year OS and PFS rates in the subgroup treated with R-CHOP therapy these significant differences vanished between two genotype groups.

The previous study in European Caucasians demonstrated no influence of *TP53* Arg72Pro on survival of DLBCL patients [15]. However, we observed better survival in patients with Arg/Arg and Arg/Pro than those with Pro/Pro, when treated with CHOP or CHOP-like therapy. Meta-analysis revealed that ethnicity may modulate the penetrance of *TP53* Arg72Pro in cancer susceptibility [9, 10]. According to the phase 3 data of 1000 Genome project, the C allele frequency is 28.53% in Europeans and 41.37% in East Asians. The C allele frequency is 25% in 205 Germany DLBCL patients and 45% in 425 Chinese DLBCL patients. Therefore, the genetic background may account for the discrepancy of clinical outcomes in two studies. Furthermore, patients in the European study were from the NHL-B1 and B2 studies, which treated aggressive lymphoma in elderly patients and good-prognosis young patients by CHOP with or without etoposide [28, 29]. However, how the 205 DLBCL patients were stratified according to good or poor prognosis and treated with or without etoposide was not clarified. In our subgroup treated with R-CHOP, the difference in survival between two genotype groups was not significant.

CHOP regimen has always the backbone of treatment strategies in DLBCL. Clinical trials had confirmed that the overall survival of patients was estimated at 50% in young and elderly patients [30–32]. However, in our study, the 5-year survival rate of patients with genotype CC (Pro/Pro) was found to be 23.2%. It is unclear exactly how *TP*53 polymorphism affects the survival to CHOP chemotherapy. Previous studies reported that when cells were exposed to doxorubicin, apoptosis was always higher in cells expressing the *TP*53 72Arg variant than those expressing the *TP*53 72Pro [33]. However, the mechanisms underlying the influence of SNP on the response to chemotherapy is still needed to further investigate in different cancers and in different populations. In general, the recombination of rituximab with CHOP therapy might be highly beneficial for Chinese patients with Pro/Pro at *TP*53 codon 72.

## Conclusion

In summary, our study revealed *TP53* Arg72 as a favorable prognostic factor in Chinese DLBCL patients treated with CHOP/CHOP-like as frontline therapy. Combination of rituximab with CHOP could optimize the survival for the Chinese patients with Pro/Pro, therefore reducing the predictive value of this biomarker with the current standard of care. This is the first report to evaluate the influence of *TP53* Arg72Pro on clinical outcomes of DLBCL patients from Asia. The prognostic implication of this SNP in other lymphoma subtypes, as well as in other cancers needs to be further studied.

## Abbreviations

CHOP: Cyclophosphamide /doxorubicin /vincristine /prednisone
CR: Complete response
Cru: Unconfirmed complete response
DLBCL: Diffuse large B-cell lymphoma
ECOG: Eastern cooperative oncology group
ESR: Erythrocyte sedimentation rate
GCB: Germinal center B cell-like
HBV: Hepatitis B virus
IPI: International prognostic index
IPI: International prognostic index
LDH: Lactate dehydrogenase
MG: Microglobulin
NHL: Non-Hodgkin lymphoma
Non-GCB: non-germinal center B cell-like
OS: Overall survival
PD: Progression disease
PFS: Progression free survival
PR: Partial response
R: Rituximab
SD: Stable disease
SNP: Single-nucleotide polymorphism

## References

[1] Dumont P, Leu JI, Della Pietra AC, George DL, Murphy M (2003). The codon 72 polymorphic variants of p53 have markedly different apoptotic potential. Nat Genet.

[2] Pim D, Banks L (2004). p53 polymorphic variants at codon 72 exert different effects on cell cycle progression. Int J Cancer.

[3] Siddique M, Sabapathy K (2006). Trp53-dependent DNA-repair is affected by the codon 72 polymorphism. Oncogene.

[4] Thomas M, Kalita A, Labrecque S, Pim D, Banks L, Matlashewski G (1999). Two polymorphic variants of wild-type p53 differ biochemically and biologically. Mol Cell Biol.

[5] Henriquez-Hernandez LA, Murias-Rosales A, Gonzalez-Hernandez A, de Leon AC, Diaz-Chico N, Fernandez-Perez L (2010). Distribution of TYMS, MTHFR, p53 and MDR1 gene polymorphisms in patients with breast cancer treated with neoadjuvant chemotherapy. Cancer Epidemiol.

[6] Ru JY, Cong Y, Kang WB, Yu L, Guo T, Zhao JN (2015). Polymorphisms in TP53 are associated with risk and survival of osteosarcoma in a Chinese population. Int J Clin Exp Pathol.

[7] Tian X, Dai SD, Sun J, Jiang SY, Jiang YH (2017). The association between the TP53 Arg72Pro polymorphism and colorectal cancer: an updated meta-analysis based on 32 studies. Oncotarget.

[8] Weich N, Ferri C, Moiraghi B, Bengio R, Giere I, Pavlovsky C, Larripa I, Fundia A (2016). TP53 codon 72 polymorphism predicts chronic myeloid leukemia susceptibility and treatment outcome. Blood Cell Mol Dis.

[9] Khan MH, Khalil A, Rashid H (2015). Evaluation of the p53 Arg72Pro polymorphism and its association with cancer risk: a HuGE review and meta-analysis. Genet Res (Camb).

[10] Francisco G, Menezes PR, Eluf-Neto J, Chammas R (2011). Arg72Pro TP53 polymorphism and cancer susceptibility: a comprehensive meta-analysis of 302 case-control studies. Int J Cancer.

[11] Kim HN, Yu L, Kim NY, Lee IK, Kim YK, Yang DH, Lee JJ, Shin MH, Park KS, Choi JS (2010). Association with TP53 codon 72 polymorphism and the risk of non-Hodgkin lymphoma. Am J Hematol.

[12] Fan C, Wei J, Yuan C, Wang X, Jiang C, Zhou C, Yang M (2014). The functional TP53 rs1042522 and MDM4 rs4245739 genetic variants contribute to non-Hodgkin lymphoma risk. PLoS One.

[13] Weng Y, Lu L, Yuan G, Guo J, Zhang Z, Xie X, Chen G, Zhang J (2012). p53 codon 72 polymorphism and hematological cancer risk: an update meta-analysis. PLoS One.

[14] Hishida A, Matsuo K, Tajima K, Ogura M, Kagami Y, Taji H, Morishima Y, Emi N, Naoe T, Hamajima N (2004). Polymorphisms of p53 Arg72Pro, p73 G4C14-to-A4T14 at exon 2 and p21 Ser31Arg and the risk of non-Hodgkin's lymphoma in Japanese. Leuk Lymphoma.

[15] Bittenbring J, Parisot F, Wabo A, Mueller M, Kerschenmeyer L, Kreuz M, Truemper L, Landt O, Menzel A, Pfreundschuh M (2008). MDM2 gene SNP309 T/G and p53 gene SNP72 G/C do not influence diffuse large B-cell non-Hodgkin lymphoma onset or survival in central European Caucasians. BMC Cancer.

[16] Wrench D, Waters R, Carlotti E, Iqbal S, Matthews J, Calaminici M, Gribben J, Lister TA, Fitzgibbon J (2009). Clinical relevance of MDM2 SNP 309 and TP53 Arg72Pro in follicular lymphoma. Haematologica.

[17] Coiffier B, Lepage E, Briere J, Herbrecht R, Tilly H, Bouabdallah R, Morel P, Van Den Neste E, Salles G, Gaulard P (2002). CHOP chemotherapy plus rituximab compared with CHOP alone in elderly patients with diffuse large-B-cell lymphoma. N Engl J Med.

[18] Cheson BD, Leonard JP (2008). Monoclonal antibody therapy for B-cell non-Hodgkin's lymphoma. N Engl J Med.

[19] Tilly H, Gomes da Silva M, Vitolo U, Jack A, Meignan M, Lopez-Guillermo A, Walewski J, Andre M, Johnson PW, Pfreundschuh M (2015). Diffuse large B-cell lymphoma (DLBCL): ESMO clinical practice guidelines for diagnosis, treatment and follow-up. Ann Oncol.

[20] Zelenetz AD, Wierda WG, Abramson JS, Advani RH, Andreadis CB, Bartlett N, Bellam N, Byrd JC, Czuczman MS, Fayad LE (2013). Non-Hodgkin's lymphomas, version 1.2013. J Natl Compr Cancer Netw.

[21] Suresh T, Lee LX, Joshi J, Barta SK (2014). New antibody approaches to lymphoma therapy. J Hematol Oncol.

[22] Bello C, Sotomayor EM. Monoclonal antibodies for B-cell lymphomas: rituximab and beyond. Hematology Am Soc Hematol Educ Program. 2007:233–42. 10.1182/asheducation-2007.1.233 18024635

[23] Elstrom RL, Martin P, Ostrow K, Barrientos J, Chadburn A, Furman R, Ruan J, Shore T, Schuster M, Cerchietti L (2010). Response to second-line therapy defines the potential for cure in patients with recurrent diffuse large B-cell lymphoma: implications for the development of novel therapeutic strategies. Clin Lymphoma Myeloma Leuk.

[24] Rimsza LM, Wright G, Schwartz M, Chan WC, Jaffe ES, Gascoyne RD, Campo E, Rosenwald A, Ott G, Cook JR (2011). Accurate classification of diffuse large B-cell lymphoma into germinal center and activated B-cell subtypes using a nuclease protection assay on formalin-fixed, paraffin-embedded tissues. Clin Cancer Res.

[25] Hans CP, Weisenburger DD, Greiner TC, Gascoyne RD, Delabie J, Ott G, Muller-Hermelink HK, Campo E, Braziel RM, Jaffe ES (2004). Confirmation of the molecular classification of diffuse large B-cell lymphoma by immunohistochemistry using a tissue microarray. Blood.

[26] Cheson BD, Pfistner B, Juweid ME, Gascoyne RD, Specht L, Horning SJ, Coiffier B, Fisher RI, Hagenbeek A, Zucca E (2007). Revised response criteria for malignant lymphoma. J Clin Oncol.

[27] Cheson BD, Horning SJ, Coiffier B, Shipp MA, Fisher RI, Connors JM, Lister TA, Vose J, Grillo-Lopez A, Hagenbeek A (1999). Report of an international workshop to standardize response criteria for non-Hodgkin's lymphomas. NCI sponsored international working group. J Clin Oncol.

[28] Pfreundschuh M, Trumper L, Kloess M, Schmits R, Feller AC, Rube C, Rudolph C, Reiser M, Hossfeld DK, Eimermacher H (2004). Two-weekly or 3-weekly CHOP chemotherapy with or without etoposide for the treatment of elderly patients with aggressive lymphomas: results of the NHL-B2 trial of the DSHNHL. Blood.

[29] Pfreundschuh M, Trumper L, Kloess M, Schmits R, Feller AC, Rudolph C, Reiser M, Hossfeld DK, Metzner B, Hasenclever D (2004). Two-weekly or 3-weekly CHOP chemotherapy with or without etoposide for the treatment of young patients with good-prognosis (normal LDH) aggressive lymphomas: results of the NHL-B1 trial of the DSHNHL. Blood.

[30] Pfreundschuh M, Kuhnt E, Trumper L, Osterborg A, Trneny M, Shepherd L, Gill DS, Walewski J, Pettengell R, Jaeger U (2011). CHOP-like chemotherapy with or without rituximab in young patients with good-prognosis diffuse large-B-cell lymphoma: 6-year results of an open-label randomised study of the MabThera international trial (MInT) group. Lancet Oncol.

[31] Pfreundschuh M, Schubert J, Ziepert M, Schmits R, Mohren M, Lengfelder E, Reiser M, Nickenig C, Clemens M, Peter N (2008). Six versus eight cycles of bi-weekly CHOP-14 with or without rituximab in elderly patients with aggressive CD20+ B-cell lymphomas: a randomised controlled trial (RICOVER-60). Lancet Oncol.

[32] Pfreundschuh M, Trumper L, Osterborg A, Pettengell R, Trneny M, Imrie K, Ma D, Gill D, Walewski J, Zinzani PL (2006). CHOP-like chemotherapy plus rituximab versus CHOP-like chemotherapy alone in young patients with good-prognosis diffuse large-B-cell lymphoma: a randomised controlled trial by the MabThera international trial (MInT) group. Lancet Oncol.

[33] Sullivan A, Syed N, Gasco M, Bergamaschi D, Trigiante G, Attard M, Hiller L, Farrell PJ, Smith P, Lu X (2004). Polymorphism in wild-type p53 modulates response to chemotherapy in vitro and in vivo. Oncogene.

